# In Vitro Assessment of Antistaphylococci, Antitumor, Immunological and Structural Characterization of Acidic Bioactive Exopolysaccharides from Marine *Bacillus* *cereus* Isolated from Saudi Arabia

**DOI:** 10.3390/metabo12020132

**Published:** 2022-02-01

**Authors:** Samy Selim, Mohammed S. Almuhayawi, Mohanned Talal Alharbi, Mohammed K. Nagshabandi, Awadh Alanazi, Mona Warrad, Nashwa Hagagy, Ahmed Ghareeb, Abdallah S. Ali

**Affiliations:** 1 Department of Clinical Laboratory Sciences, College of Applied Medical Sciences, Jouf University, Sakaka 72341, Saudi Arabia; aaalanazi@ju.edu.sa; 2 Department of Medical Microbiology and Parasitology, Faculty of Medicine, King Abdulaziz University, Jeddah 21589, Saudi Arabia; msalmuhayawi@kau.edu.sa (M.S.A.); 3 Department of Medical Microbiology and Parasitology, Faculty of Medicine, University of Jeddah, Jeddah 23218, Saudi Arabia; mtalharbi@uj.edu.sa (M.T.A.); mknagshabandi@uj.edu.sa (M.K.N.); 4 Department of Clinical Laboratory Sciences, College of Applied Medical Sciences at Al-Quriat, Jouf University, Al-Quriat 77454, Saudi Arabia; mfwarad@ju.edu.sa; 5 Department of Biology, College of Science and Arts at Khulis, University of Jeddah, Jeddah 21959, Saudi Arabia; niibrahem@uj.edu.sa; 6 Botany and Microbiology Department, Faculty of Science, Suez Canal University, Ismailia 41522, Egypt; 7 Botany and Microbiology Department, Faculty of Science, Cairo University, Giza 12613, Egypt; ahmed.ghareeb@med.asu.edu.eg; 8 Department of Microbiology, Faculty of Agriculture, Cairo University, Giza 12613, Egypt

**Keywords:** Antistaphylococci, MRSA, antioxidant, anti-inflammatory, antitumor, Exopolysaccharide, *Bacillus cereus*

## Abstract

A strain of *Bacillus cereus* was isolated from the Saudi Red Sea coast and identified based on culture features, biochemical characteristics, and phylogenetic analysis of 16S rRNA sequences. EPSR3 was a major fraction of exopolysaccharides (EP_S_) containing no sulfate and had uronic acid (28.7%). The monosaccharide composition of these fractions is composed of glucose, galacturonic acid, and arabinose with a molar ratio of 2.0: 0.8: 1.0, respectively. EPSR3 was subjected to antioxidant, antitumor, and anti-inflammatory activities. The results revealed that the whole antioxidant activity was 90.4 ± 1.6% at 1500 µg/mL after 120 min. So, the IC_50_ value against DPPH radical found about 500 µg/mL after 60 min. While using H_2_O_2_, the scavenging activity was 75.1 ± 1.9% at 1500 µg/mL after 60 min. The IC_50_ value against H_2_O_2_ radical found about 1500 µg/mL after 15 min. EPSR3 anticytotoxic effect on the proliferation of (Bladder carcinoma cell line) (T-24), (human breast carcinoma cell line) (MCF-7), and (human prostate carcinoma cell line) (PC-3) cells. The calculated IC50 for cell line T-24 was 121 ± 4.1 µg/mL, while the IC_50_ for cell line MCF-7 was 55.7 ± 2.3 µg/mL, and PC-3 was 61.4 ± 2.6 µg/mL. Anti-inflammatory activity was determined for EPSR3 using different methods as Lipoxygenase (LOX) inhibitory assay gave IC_50_ 12.9 ± 1.3 µg/mL. While cyclooxygenase (COX-2) inhibitory test showed 29.6 ± 0.89 µg /mL. EPSR3 showed potent inhibitory activity against methicillin-resistant *Staphylococcus aureus* (MRSA) and coagulase-negative staphylococci. The exposure times of EPSR3 for the complete inhibition of cell viability of methicillin resistant *S. aureus* was found to be 5% at 60 min. Membrane stabilization inhibitory gave 35.4 ± 0.67 µg/mL. EPSR3 has antitumor activity with a reasonable margin of safety. The antitumor activity of EPSR3 may be attributed to its content from uronic acids with potential for cellular antioxidant and anticancer functional properties.

## 1. Introduction

Bacterial exopolysaccharides are extracellular organic macromolecules that play a significant role in various bacterial cellular activities, including phage protection, bacterial cell clustering under osmatic stress, and surface adhesion [1]. The exopolysaccharides (EPS) is made up of biofilm structural components engaged in preserving water and processing foreign organic compounds and inorganic ions to prevent cell desiccation [2].

pH, yeast, and sucrose extract supply are factors that impact the synthesis and generation of EPS [3]. By adjusting the different parameters, the EPS generating yield may be increased. The most well-known producers of EPS are *Lactobacillus, Lactococcus, Bifidobacterium, Leuconostoc, Pediococcus, Streptococcus, Enterococcus* and *Weissella sp.* [4]. Si et al. [5] investigated the EPS production of *Lactobacillus plantarum* YM-2, finding 24 mg/L prior to optimization and 257 mg/L after optimization.

Drug delivery, medicinal coatings, surgical sealants, and anticancer activities are just a few of the medical and industrial applications for EPS [6]. *Aerococcus, Carnobacterium, Enterococcus, Lactobacillus, Lactococcus, Leuconostoc, Oenococcus, Pediococcus, Streptococcus,* and *Weissella,* were reported to leave a significant impact in rapid industrialization [7].

EPS has also been used as a powerful antioxidant agent due to its bioavailability in nature. Because of its vast spectrum of biological uses, there has been an increasing interest in discovering EPS in recent years [8]. The EPS generated by *Lactobacillus acidophilus, Lactobacillus gasseri*, *Lactobacillus plantarum*, and *Lactobacillus rhamnosus* isolated from diverse sources have been shown to exhibit antitumor as well as antioxidant properties [9,10].

Microorganisms that produced antibiotics and enzymes from harsh environments showed great promise in biotechnological applications [11,12]. Numerous studies have been conducted on the growth of bacterial EPS, which comprises larger levels of nucleic acids, proteins, and polysaccharides [13].

Bacterial strains that have been found to have a high affinity for EPS production is critical. Furthermore, the EPS production by Gram-positive Bacillus species has been proven, and they are currently considered prospective antioxidant agents [1].

As far as we know, no research studies have been published concerning the isolation, identification, or generation of EPS from bacteria obtained from marine sediment sources from the Red Sea, Saudi Arabia. Therefore, this study aims to isolate and biochemically characterize EPS isolated from marine *B. cereus* and, further, to assay its in vitro anti-staphylococci, antioxidant; anti-inflammatory; antitumor; and immunological activities.

## 2. Results

### 2.1. Isolation and Identification of the EPS Producing Bacteria

For the screening program to produce EPSs, five bacterial isolates were isolated from a marine source based on their unique morphological features colony. Two strains were discovered to be producers of EPSs. One of these marine bacteria isolated from the sea produced the most EPS (R3) (7.95 g/L). Standard morphological, physiological, and biochemical plates revealed that strain R3 was a Gram-positive bacillus with a dull colony surface, an undulate edge, a large irregular colony, and no pigments. A Catalase test, Voges-Proskauer test, Simon citrate test and Nitrate reduction all showed a positive promising yielded.

**Figure 1:**
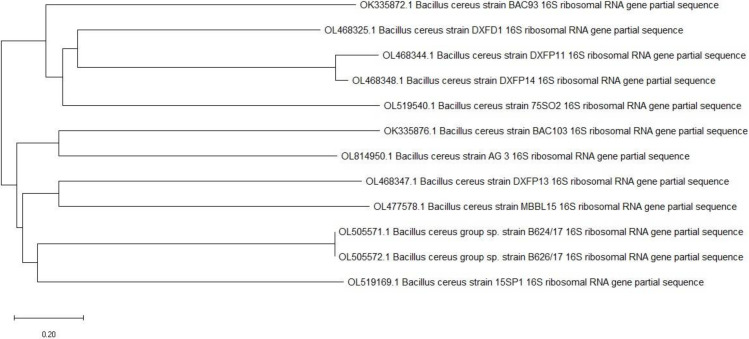
16S rRNA of the local isolate *Bacillus cereus* strain AG 3 respects to closely related sequences available in Gen Bank databases.

The nucleotide sequence has been entered into the GenBank database. So, it was identified as *Bacillus cereus* strain AG3 with accession number OL814950 (Figure 1). 

### 2.2. Isolation, Partial Purification and Composition of EPSR3

Exopolysaccharide production reached a maximum of 7.95 g/L, subjected to partial purification and fractionation by being redissolved in deionized water followed by dialysis in contradiction of deionized water for 72 h. The dialyzed solution was precipitated by 1, 2, 3, and 4 volumes of absolute cold ethanol. The main fraction of EPSR3 (85%) was obtained after fractionated with three volumes of ethanol precipitation from the crude EPS. It was yellow, odorless powder, and soluble in water but insoluble in ethanol and other organic solvents.

There was no sulfate in the EPSR3 fraction, although it did contain uronic acid (28.7%). The monosaccharide composition of this fraction is 2.0: 0.8: 1.0, with glucose, galacturonic acid, and arabinose being the molar ratios. This suggests that this fraction is an acidic heteropolysaccharide. GPC looked at EPSR3 weight average molecular weight (M_w_), number average of molecular weights (M_n_), and polydispersity (M_w_/M_n_). In the GPC chromatogram, the EPSR3 molecules were widely scattered, with a polydispersity index (*PI*) of 1.2 and an overall average molecular weight (M_w_) of 1.66 × 10^4^ g/mol and a number average molecular weight (M_n_) 1.37 × 10^4^ g/mol.

**Figure 2:**
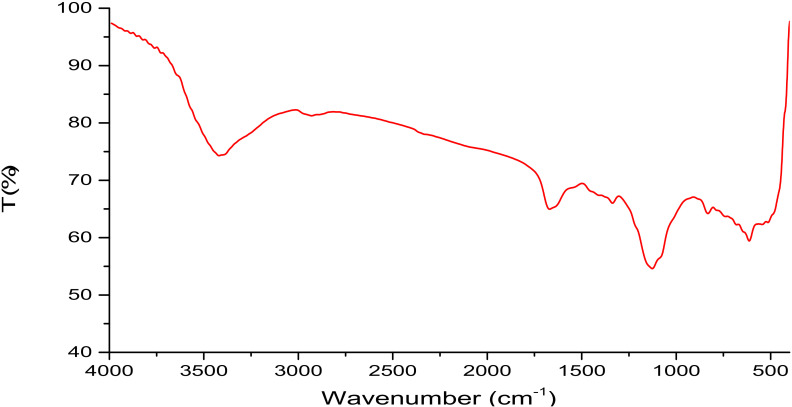
FTIR Spectrum of EPSR3.

The FTIR spectrum fraction exhibited a significant as showed in Figure 2, broad characteristic peak at around at 3420.14 cm−^1^ region was attributed to the expansion vibration of O–H in the ingredient sugar residues. The EPSR3 fraction also has a band at 1670.05 cm^−1^, dominated by circle vibrations. The band at 1126.22 cm^−1^ indicated the SO = 3. interfered with stretching vibration of C-O glycosidic bond vibration, and the strap at 832.13 cm^−1^ suggested the β-pyranose.

### 2.3. Antioxidant Activity of EPSR3

**Figure 3:**
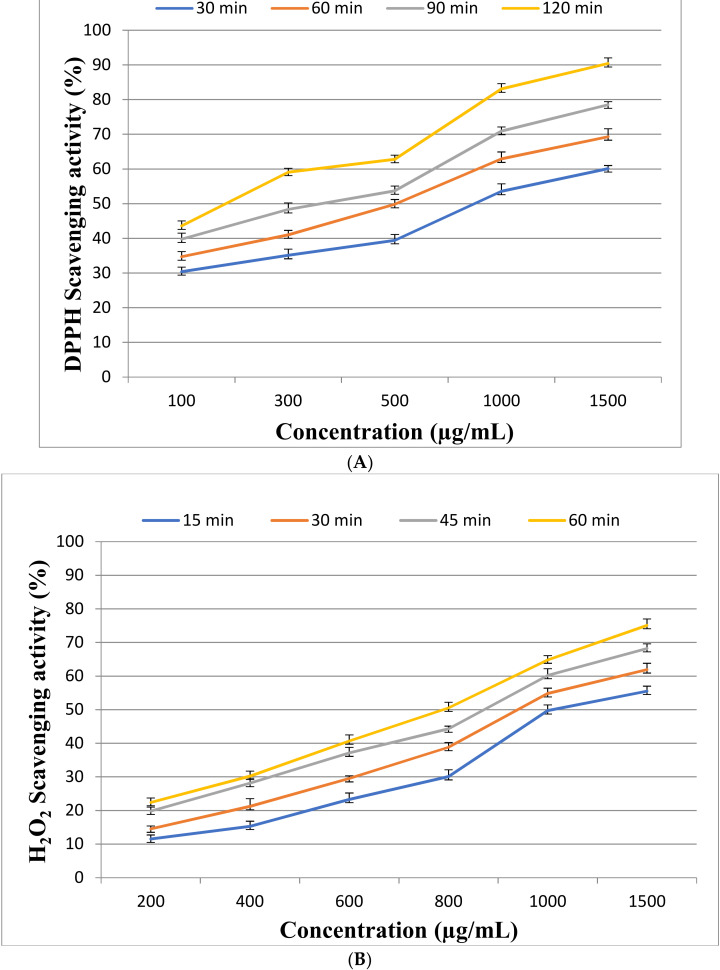
Scavenging activity of EPSR3 at different times (**A**) DPPH and (**B**): H_2_O_2._

At different intervals, the antioxidant activity was measured quantitatively at (30, 60, 90, and 120 min). Figure 3A shows that by increasing EPSR3 concentrations from 100, 300, 500, 1000, and 1500 g/mL, the overall antioxidant activity is enhanced. Maximum antioxidant activity was 90.4 ± 1.6% at 1500 µg/mL after 120 min. So, the IC_50_ value against DPPH radical found about 500 µg/mL after 60 min. While using H_2_O_2_ scavenging activity to evaluate EPSR3’s capacity to scavenge hydrogen peroxide at various concentrations (200, 400, 600, 800, 1000, and 1500 g/mL), the highest extreme activity was 75.1 ± 1.9% at 1500 µg/mL after 60 min. The IC_50_ value against H_2_O_2_ radical found about 1500 µg/mL after 15 min (Figure 3B).

### 2.4. Antitumor Activity against Different Cell Lines

**Figure 4:**
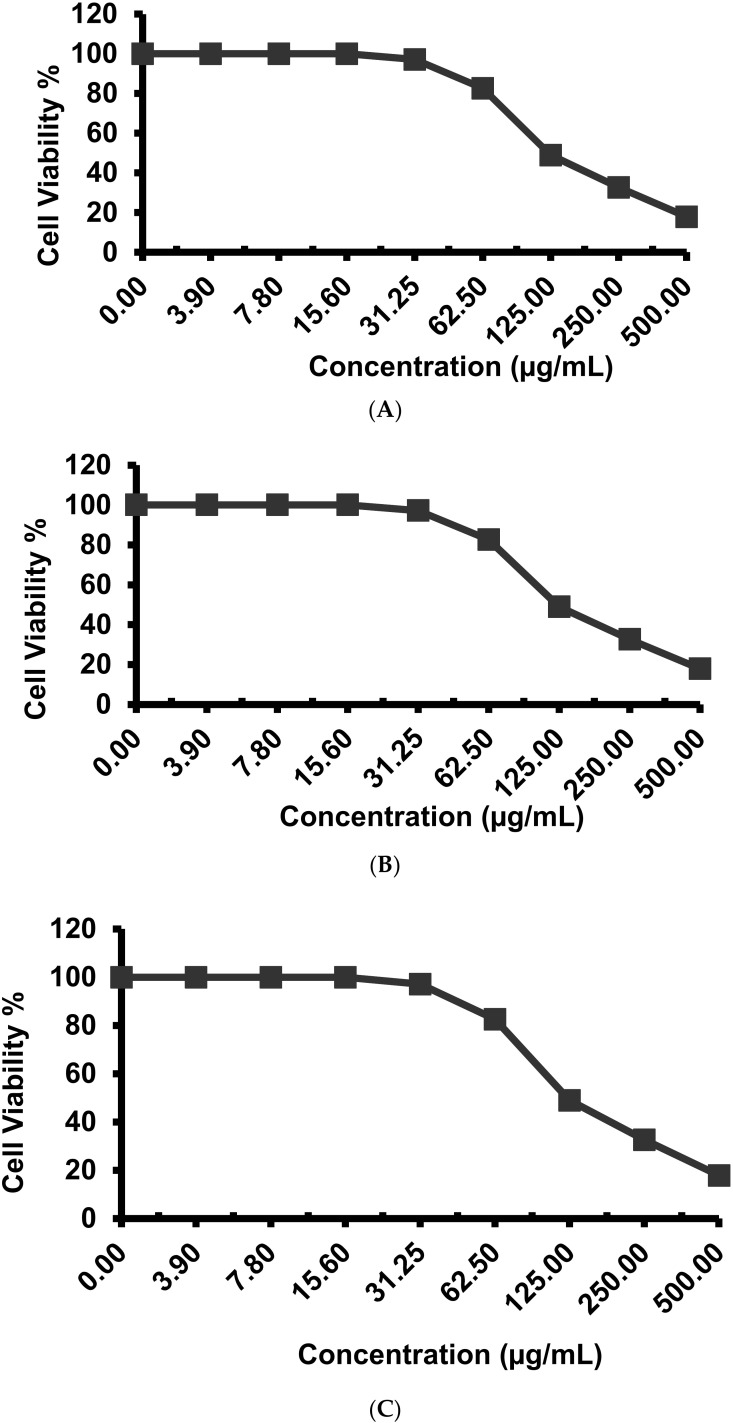
Evaluation of cytotoxicity against different cell line (**A**): T-24 and (**B**): MCF7 and (**C**) PC-3.

As shown in Figure 4, EPSR3 influenced the proliferation of T-24, MCF-7, and PC-3 cells. The determined IC_50_ for cell line T-24 was 121 ± 4.1 µg/mL, whereas the IC_50_ for cell lines MCF-7 and PC-3 was 55.7 ± 2.3 µg/mL and 61.4 ± 2.6 µg/mL, respectively.

### 2.5. Anti-Inflammatory Activity

**Figure 5:**
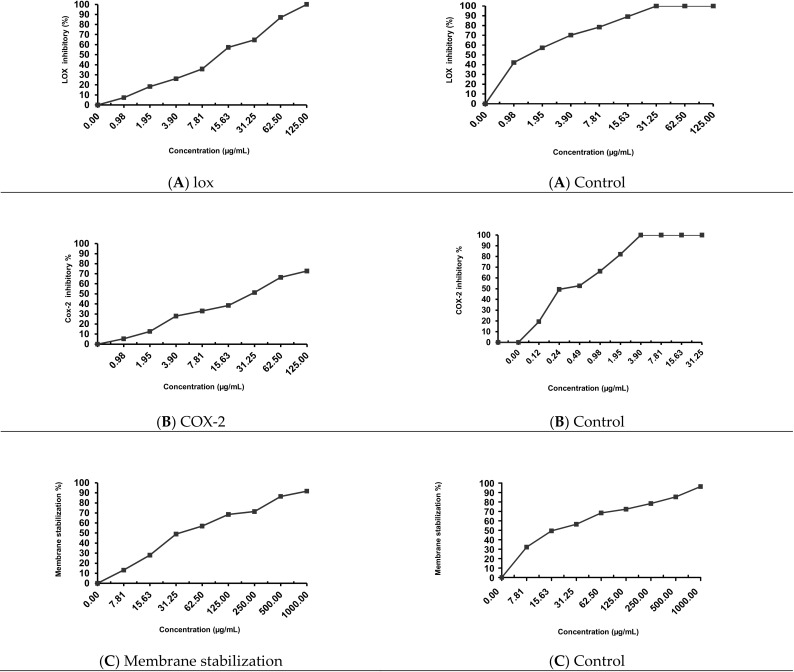
Evaluation of anti-inflammatory activity against different cell line (**A**): LOX; (**B**): COX-2 and (**C**) Membrane stabilization.

EPSR3 anti-inflammatory activity was assessed using various methods, including the Lipoxygenase (LOX) inhibitory as shown in Figure 5A, which had an IC_50_ 12.9 ± 1.3 µg/mL. In contrast, the control sample (ibuprofen) had an IC_50_ 1.5 ± 1.3 µg/mL. Therefore, the COX-2 inhibitory shown in Figure 5B gave 29.6 ± 0.89 µg /mL, while control (Celecoxib) gave 0.28 ± 1.7 µg/mL. Figure 5C shows that membrane stabilization inhibitory gave 35.4 ± 0.67 µg/mL. In comparison, control (Indomethacin) is 17.02 ± 0.82 µg/mL.

**Figure 6:**
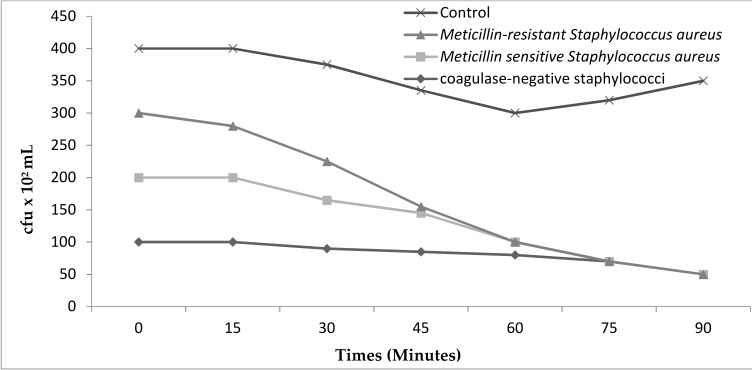
Effect of EPSR3 (5%) on the viability of Methicillin–resistant *Staphylococcus aureus* (MRSA), Methicillin–senstive *Staphylococcus aureus* (MSSA) and coagulase- negative Staphylococci. Values are the average of three individual replicates (means ± S.D). Differences between samples were determined by the Student’s t-test and were considered to be significant when *p* ≤ 0.05.

EPSR3 showed potent inhibitory activity (MIC/MBC: 0.5/2 mg/mL) against *S. aureus*. The MIC/MBC values of the isolated compounds against methicillin-resistant *S. aureus* (MRSA),methicillin-sensitive *S. aureus* and coagulase-negative staphylococci. The MICs and MBCs for the 12 isolates, as determined by the broth micro dilution method. EPSR3 compounds showed very potent inhibitory activity against clinical isolates of *S. aureus*. The effect on the cell viabilities of *S. aureus* demonstrated that exposure of date extract at 5% concentration had a potential antibacterial impact on the viabilities of strains. The exposure times of EPSR3 for the complete inhibition of cell viability of methicillin resistant *S. aureus* was found to be 5% at 60 min (Figure 6). 

## 3. Discussion

Different bacteria genera generate EPS, and Bacillus spp. have been found to create extracellular polysaccharides [14]. Although the antioxidant capabilities of EPS from *Bacillus* spp. probiotic bacteria have been demonstrated [15], the antioxidant activities of marine *Bacillus* spp. have yet to be fully investigated. We studied the antioxidant, anti-inflammatory, and anticytotoxic effects of EPS from *B. cereus* AG3 isolated from a marine sediment source from the Red Sea as part of our search for novel bioactive metabolites from marine microorganisms and the antioxidant potential of bacterial extracts.

Two bacterial isolates were discovered as producing EPSs out of five examined, with the greatest production coming from a marine bacterium isolate (R3). Chemical examination of pure EPSR3 revealed no sulfate and the presence of uronic acid. Furthermore, the monosaccharide composition of these fractions was revealed, with glucose, galacturonic acid, and arabinose being the molar ratios. This indicates that the portion in question is an acidic heteropolysaccharide.

It is worth noting that, while most EPS’ fundamental carbohydrate structures remain relatively constant, the concentration of their substituent groups can fluctuate, affecting the EPS’ properties and activity [16].

The overall average molecular weight (Mw) of EPSR3 in the GPC chromatogram was 1.66 × 10^4^ g/mol and number average molecular weight (Mn) of 1.37 × 10^4^ g/mol. Therefore, it is worth noting that EPSR3 has a high molecular weight. These findings are consistent with a prior study that suggested Mw of EPSs ranging from 10 and 6000 kDa [17]. The expansion vibration of O–H in the component sugar residues were linked to a substantial broad characteristic peak in the FTIR spectrum fraction, as seen in Figure (4), at about 3420.14 cm^−1^ [18]. The EPSR3 fraction also seems to contain a band at 1670.05 cm^−1^ that is dominated by circular vibrations [19]. The band at 1126.22 cm^−1^ indicated the SO = 3 interfered with stretching vibration of C-O glycosidic bond vibration, and the strap at 832.13 cm1 pointed the pyranose [20].

Few papers have been published on *Bacillus* strains that produce EPS and its antioxidant capabilities. The bioflocculant EPS was generated by a soil *Bacillus subtilis* strain [21]. *Bacillus* EPS production has been discovered to be potentially effective in removing dangerous heavy metal pollutants from sewage treatment systems [22]. The antioxidant and free radical scavenging activities of the EPS of four different monosaccharides produced by *B. coagulans* (glucose, fructose, galactose, and mannose) were investigated [23]. This is the first time EPS from marine *Bacillus* has been demonstrated to scavenge superoxide and hydroxyl radicals in a concentration-dependent manner, with IC_50_ value of about 500 µg/mL after 60 min against DPPH radical found and maximum antioxidant activity 90.4 ± 1.6% at 1500 µg/mL after 120 min.

The hydroxyl group, as well as the presence of uronic acid, are responsible for EPSR3’s antioxidant action (28.7%). Ye et al. [24] isolated and purified an acidic EPS from marine *Pseudomonas* PF-6 that belongs to the β-type heteropolysaccharide and possesses a pyran group that exhibited antioxidant activity. Additionally, [25] in another report EPS isolated from *Bacillus amyloliquefaciens* 3MS 2017 may also scavenge DPPH free radicals with a maximal activity of 99.39% at 1000 g/mL. *Streptomyces carpaticus* yielded an EPS with an EC_50_ value of 111 g/mL that has DPPH antioxidant activity, as reported by [26].

Compositionally, EPSR3 consisted of three different monosaccharides, including glucose, galacturonic acid, and arabinose. Except for glucuronic acid, these monosaccharides are efficient reductive agents since they have an aldehyde group [27]. The radical scavenging capacity of EPS may be due to the reductive capacity of such monosaccharides. Purified polysaccharides generated from crude polysaccharides have been more functional in vitro compared to crude polysaccharides in several studies [28].

The high purity of EPS must be guaranteed, and the purity must be accurately recorded. The anticancer benefits of EPS are frequently misunderstood because of the unexpected and ambiguous impacts of the undesired components. Taking into consideration that the purity of the extracted EPS is affected by differences in isolation and purification procedures, more investigation into the purification of crude EPS from marine *Bacillus cereus* AG3 and the refined fractions’ molecular structure and antioxidant capabilities is required. 

Next, as shown in Figure 4, the effect of EPSR3 on the proliferation of T-24, MCF-7, and PC-3 cells is evaluated. EPS from *L. plantarum*, *L. acidophilus*, and *L. helveticus* are the most commonly reported EPS with good anticancer properties among EPS-producing species. Even from the same species, the antiproliferative activity of EPS differed from strain to strain [9,29]. The apoptotic anticytotoxic activity of EPS has recently been reported [30]. However, we would like to shed some light on the contentious influence of Mw on the anticancer function of EPS.

According to various studies, EPS with a high Mw are more effective against cancer since they cannot enter the cell and instead connect with cancer cell receptors that govern signaling and transduction [31,32,33,34]. Others, on the other hand, felt that low Mw permitted EPS to penetrate past the cell membrane barrier more efficiently, allowing it to fulfill biological activities such as cell cycle arrest [35]. In our finding, molecular weight (Mw) of 1.66 × 104 g/mol and number average molecular weight (Mn) of 1.37 × 104 g/mol was calculated.

Furthermore, the inclusion of specific structures such as uronic acid, sulfate [36,37], β-type glycosidic linkages [37], protein molecules [38] and side chains [39] may influence the EPS’ anticancer activity. The current EPSR3 contained no sulfate but uronic acid (28.7%).

It is consequently suggested that acetylation, phosphorylation, carboxymethylation, and sulfonation be used to improve such cytotoxic activities [36,40]. Another important point to mention is that the microbiological source and tumor cells targeted appear to make a difference in the anticancer effects of EPS. However, no conclusions can be reached based on current research on which bacterial source has the most impact or which type of tumor cell is the most responsive. These characteristics contribute to a considerable rise in the incompatibility of research. Furthermore, as previously indicated, the wide range of extraction and isolation processes adds to the incomparability.

Finally, EPSR3 anti-inflammatory activity was assessed using various methods, including the Lipoxygenase (LOX) inhibitory, as shown in Figure 5. This anti-inflammatory effect can be related to its structure and the inhibitory activity of cyclooxygenases [41]. Furthermore, it is thought that exopolysaccharide’s principal effect is to modulate cytokines and their related transcription factors [42]. TNF-α, IL-1, and IL-6, which are pro-inflammatory cytokines, and IL-10, which is an anti-inflammatory cytokine, are the primary mediators of these natural products’ effects [43].

## 4. Materials and Methods

### 4.1. Sampling and Isolation of Bacteria

Samples were collected from the Red Sea’s marine sediment sources in Saudi Arabia. The serial dilution approach was used to isolate bacteria [44] on marine media. The following ingredients were dissolved in 750 mL seawater to make 1 L: glucose 20, CaCO_3_ 1.0, NH_4_NO_3_ 0.8, KH_2_PO_4_ 0.05, K_2_HPO_4_ 0.6, MgSO_4_.7H_2_O 0.05, MnSO_4_. 4H_2_O 0.1, yeast extract 0.1 [45].

### 4.2. Identification of Bacterial Isolates

Based on physical and biochemical parameters, the isolate that generated the most EPSs and had the highest antioxidant activity was determined [46]. Phylogenic analysis was used to validate the identification [47]. On a 1.2 percent agarose gel, genomic DNA from the bacterial isolate was extracted, and the quality was assessed; a single band of high Mw DNA was detected. The forward primer was 5′-TCCGTAGGTGAACTTTGCGG-3′, and the reverse primer was 5′-TCCTCCGCTTATTGATATGC-3′ PCR [48]. The DNA sequence was compared to the GenBank database at the National Center for Biotechnology Information (https://www.ncbi.nlm.nih.gov/ (accessed on 29 December 2021)) using the BLAST tool. The phylogenetic tree was created by aligning sequences with the highest similarity to the 16S rRNA sequences of the bacterial isolate. The bacteria’s 16S rRNA gene sequences were submitted to the DDBJ/EMBL/GenBank nucleotide sequence databases.

### 4.3. Production and Fractionation of EPS

The promising strain (R3) was chosen for EPS production. The production medium’s fermented broth contained (g/L) sucrose 20, yeast extract 2, and peptone 4, dissolved in 750 mL seawater and diluted to 1 L to remove bacterium cells. The sample was collected and centrifuged at 4000 rpm for 30 min at 4 °C. TCA (10%) was added and kept overnight at 4 °C before centrifuging for 20 min at 5000 rpm to remove protein. With NaOH solution, the pH of the supernatant was adjusted to 7 [49]. The bacterial mass was collected by centrifugation after four liters of absolute cold ethanol were added to the supernatant. The residue was redissolved in deionized water, and then dialysis was performed for 72 h against deionized water. Fractional precipitation was performed on the dialyzed solution using 1, 2, 3, and 4 L of absolute cold ethanol, respectively. To check the presence of proteins and nucleic acids, the UV absorption spectra were collected between 200 and 800 nm [50].

### 4.4. Analysis of EPSR3

EPSR3 FTIR spectra were obtained using KBr pellets (2.0 mg sample and 200 mg KBr, respectively, using the FTIR-UNIT Bruker Vector 22 Spectrophotometer), as described in the paper [51]. The m-hydroxybiphenyl colorimetric technique was used to identify uronic acids at 525 nm [52]. The turbidity technique determined sulfate [53]. The monosaccharide composition was defined (Agilate Pack, serics1, 200), using an Aminex carbohydrate HP-87C column (300 × 7.8 mm) and 0.5 mL/min deionized water as the mobile phase [54]. High-performance chromatography (HPLC, Agilent 1100 Series System, Hewlett-Packard, Germany) with refractive index (RI) detection was used to measure the average molecular weight (Mw). The M_w_/M_n_ ratio was used to construct the polydispersity index (PI) [55].

### 4.5. Assessment of Antioxidant Activity 

#### 4.5.1. DPPH Assay

EPSR3 at a varied concentration of 100, 300, 500, 1000, and 1500 g/mL was utilized in a DPPH experiment with a combination of 2 mL DPPH solution and EPSR3 to determine antioxidant activity. The mixture was aggressively shaken and permitted to stand in the dark for 30, 60, 90, and 120 min, with the absorbance measured at 517 nm, Brand-Williams et al. [56], and the scavenging activity computed as follows:

Scavenging ability (%) = (A _control_−A _sample /_A _control_) × 100

#### 4.5.2. Hydrogen Peroxide Scavenging (H_2_O_2_) Assay

EPSR3’s capacity to scavenge H_2_O_2_ was evaluated according to Ruch et al. [57]. EPSR3 was added to H_2_O_2_ at various concentrations (200, 400, 600, 800, 1000, and 1500 g/mL) in distilled H_2_O, and absorbance was measured at 230 nm. The following formula computed the percentage of H_2_O_2_ scavenging:

Scavenging ability (%) = (A _control_−A _sample /_A _control_) × 100

### 4.6. Evaluation of Cytotoxic Effects Using Different Cell Line 

For cytotoxicity assay, the cells of MCF-7 cells (human breast carcinoma cell line), PC-3 cells (human prostate carcinoma cells), and T-24 (Bladder carcinoma) were seeded in a 96-well plate at a cell concentration of 1 × 10^4^ cells per well in 100 µL of growth medium. The percentage of viability was calculated as [(ODt/ODc)] × 100%, where ODt is the mean optical density of wells treated with the tested sample and ODc is the mean optical density of untreated cells [58]. 

### 4.7. Evaluation of Anti-Inflammatory Activity

#### 4.7.1. In Vitro Lipoxygenase (LOX) Inhibition

EPSR3 and the reference compound (Ibuprofen) were tested to examine the anti-inflammatory response by inhibiting the LOX enzyme from Glycine max (type I-B). This assay was performed according to Granica et al. [59]. The inhibitory percentages were calculated according to the formula:

Inhibitory activity (%) = (1 − As/Ac) × 100, where As is the absorbance in the presence of test substance and Ac is the absorbance of control.

IC_50_ values—the inhibitory concentration of the samples required to decrease the enzyme’s activity by 50%—were determined from the plotted graphs of enzyme inhibition (%) against the concentrations of the samples.

#### 4.7.2. In Vitro Cyclooxygenase (COX-2) Inhibition

EPSR3 was evaluated at concentrations ranging from 125 to 0.98 g/mL to assess the anti-inflammatory response induced by inhibiting the COX-2 enzyme. With minor adjustments, this experiment was carried out according to [60,61]. The inhibitory activity was evaluated using a microplate reader to detect the rise in absorbance at 611 nm (BIOTEK; Santa Clara, CA, USA). The inhibitory percentages were determined using the following formula: Inhibitory activity (percent) = (1 − As/Ac) × 100, whereas is the absorbance when the test drug is present, and Ac is the absorbance when the control substance is present. The concentration inducing 50% enzyme inhibition was used to measure the effectiveness of extracts and the reference chemical (Celecoxib) in inhibiting the COX-2 isoenzyme (IC_50_).

#### 4.7.3. Membrane Stabilization

The membrane stabilizing activity of EPSR3 was assessed using hypotonic solution-induced erythrocyte hemolysis, according to [62]. The percentage inhibition of hemolysis or membrane stabilization was calculated by:

% Inhibition of hemolysis (membrane stabilization %) = 100 × {OD1 − OD2/OD1} Where: OD1 = Optical density of hypotonic-buffered saline solution alone OD2 = Optical density of test sample in hypotonic solution. The IC_50_ value was defined as the concentration of the sample to inhibit 50% RBCs hemolysis under the assay conditions.

### 4.8. Antimicrobial Tests

#### 4.8.1. Microbial Strains 

The agar diffusion assay was performed according to a modified Kirby–Bauer disc diffusion method. One loopful of each test organism was suspended in 3 mL 0.9% NaCl solution separately. All clinical isolates of methicillin resistant and sensitive *Staphylococcus aureus* and coagulase-negative staphylococci were isolated from human beings and belong to the microbiological laboratory collection of the microbiology laboratory of Department of Clinical Laboratory Sciences, College of Applied Medical Sciences, Jouf University, Saudi Arabia. Identification and antimicrobial susceptibility of the isolated strains were performed by a VITEK automated system (BioMerieux, Marcy I’Etoile, France). Nutrient agar was inoculated with this suspension of the respective organism and poured into a sterile petri dish. 

#### 4.8.2. Disc-Diffusion Assay

The EPSR3 were dissolved in dimethylsulfoxide (DMSO) to a final concentration of 30 mg/mL and sterilized by filtration by 0.45 µm Millipore filters. Antistaphylococci tests were then carried out by disc diffusion method [63] using 100 μL of suspension containing 10^8^ cfu/mL of bacteria spread on nutrient agar (NA). The discs (6 mm in diameter) were impregnated with 5 mg/disc and placed on the inoculated agar. Negative controls were prepared using the same solvent employed to dissolve extract. Oxicillin (30 μg/disc) was used as positive reference standards to determine the sensitivity of one strain/isolate in each microbial species tested. The inoculated plates were incubated at 37 °C for 24 h for clinical bacterial strains. Antimicrobial activity was evaluated by measuring the zone of inhibition against the test organisms.

#### 4.8.3. Statistical Analysis 

The variations between experiments were estimated by standard deviations, and the statistical significance of changes was estimated using the student’s *t*-test. Only the probability P ≤ 5% was regarded as indicative of statistical significance.

## 5. Conclusions

In this study, a marine *Bacillus* strain isolated from the Red Sea Saudi coast produced EPSR3 at (7.95 g/L). From 16S rRNA analysis, the strain was related to *B. cereus* strain AG3. The EPS displayed moderate scavenging activity on superoxide, hydroxyl radical, and DPPH radicals. In addition, the EPS exhibited a significant protective impact on cancer cell lines. Based on the chemical analysis and in vitro assessments, this study suggests that the EPS from marine *B. cereus* might contribute to a potential application as a natural antioxidant agent and a new therapeutic agent for treatment of staphylococcal infection and cancers diseases. 

## Data Availability

Data available in a publicly accessible repository. The data presented in this study are openly available in DDBJ/EMBL/GenBank nucleotide sequence databases at https://www.ncbi.nlm.nih.gov/nuccore/2163748932, reference number GenBank: OL814950.1.

## References

[1] Angelin J., Kavitha M. (2020). Exopolysaccharides from Probiotic Bacteria and Their Health Potential. Int. J. Biol. Macromol..

[2] Flemming H.-C., Neu T., Wingender J. (2016). The Perfect Slime: Microbial Extracellular Polymeric Substances (EPS). Water Intell. Online.

[3] Kuntiya A., Hanmoungjai P., Techapun C., Sasaki K., Seesuriyachan P. (2010). Influence of PH, Sucrose Concentration and Agitation Speed on Exopolysaccharide Production by Lactobacillus Confusus TISTR 1498 Using Coconut Water as a Raw Material Substitute. Maejo Int. J. Sci..

[4] Prete R., Alam M.K., Perpetuini G., Perla C., Pittia P., Corsetti A. (2021). Lactic Acid Bacteria Exopolysaccharides Producers: A Sustainable Tool for Functional Foods. Foods.

[5] Si T., Chenjian L., Qin X., Li X., Luo Y., Yang E. (2017). Optimization of Biosynthesis Conditions for the Production of Exopolysaccharides by Lactobacillus Plantarum YM-2. Food Sci..

[6] Mohd Nadzir M., Nurhayati R.W., Idris F.N., Nguyen M.H. (2021). Biomedical Applications of Bacterial Exopolysaccharides: A Review. Polymers.

[7] De Souza de Azevedo P.O., Mendonça C.M.N., Moreno A.C.R., Bueno A.V.I., de Almeida S.R.Y., Seibert L., Converti A., Watanabe I.-S., Gierus M., de Souza Oliveira R.P. (2020). Antibacterial and Antifungal Activity of Crude and Freeze-Dried Bacteriocin-like Inhibitory Substance Produced by Pediococcus Pentosaceus. Sci. Rep..

[8] Escárcega-González C.E., Garza-Cervantes J.A., Vázquez-Rodríguez A., Morones-Ramírez J.R. (2018). Bacterial Exopolysaccharides as Reducing and/or Stabilizing Agents during Synthesis of Metal Nanoparticles with Biomedical Applications. Int. J. Polym. Sci..

[9] Sungur T., Aslim B., Karaaslan C., Aktas B. (2017). Impact of Exopolysaccharides (EPSs) of Lactobacillus Gasseri Strains Isolated from Human Vagina on Cervical Tumor Cells (HeLa). Anaerobe.

[10] Adesulu-Dahunsi A.T., Jeyaram K., Sanni A.I., Banwo K. (2018). Production of Exopolysaccharide by Strains of Lactobacillus Plantarum YO175 and OF101 Isolated from Traditional Fermented Cereal Beverage. PeerJ.

[11] Adrio J.L., Demain A.L. (2014). Microbial Enzymes: Tools for Biotechnological Processes. Biomolecules.

[12] Coker J.A. (2016). Extremophiles and Biotechnology: Current Uses and Prospects. F1000Research.

[13] Costa O.Y.A., Raaijmakers J.M., Kuramae E.E. (2018). Microbial Extracellular Polymeric Substances: Ecological Function and Impact on Soil Aggregation. Front. Microbiol..

[14] Vijayabaskar P., Babinastarlin S., Shankar T., Sivakumar T., Anandapandian K.T.K. (2011). Quantification and Characterization of Exopolysaccharides from Bacillus Subtilis (MTCC 121). Adv. Biol. Res..

[15] Kodali V.P., Perali R.S., Sen R. (2011). Purification and Partial Elucidation of the Structure of an Antioxidant Carbohydrate Biopolymer from the Probiotic Bacterium Bacillus Coagulans RK-02. J. Nat. Prod..

[16] Casillo A., Lanzetta R., Parrilli M., Corsaro M.M. (2018). Exopolysaccharides from Marine and Marine Extremophilic Bacteria: Structures, Properties, Ecological Roles and Applications. Mar. Drugs.

[17] Xie J.-H., Xie M.-Y., Nie S.-P., Shen M.-Y., Wang Y.-X., Li C. (2010). Isolation, Chemical Composition and Antioxidant Activities of a Water-Soluble Polysaccharide from Cyclocarya Paliurus (Batal.) Iljinskaja. Food Chem..

[18] Kanmani P., Kumar R.S., Yuvaraj N., Paari K.A., Pattukumar V., Arul V. (2011). Production and Purification of a Novel Exopolysaccharide from Lactic Acid Bacterium Streptococcus Phocae PI80 and Its Functional Characteristics Activity in Vitro. Bioresour. Technol..

[19] Sun R., Fang J., Goodwin A., Lawther J., Bolton A.J. (1998). Fractionation and Characterization of Polysaccharides from *Abaca Fibre*. Carbohydr. Polym..

[20] Cheng A., Wan F., Jin Z., Wang J., Xu X. (2008). Nitrite Oxide and Inducible Nitric Oxide Synthase Were Regulated by Polysaccharides Isolated from Glycyrrhiza Uralensis Fisch. J. Ethnopharmacol..

[21] Patil S.V., Bathe G.A., Patil A.V., Patil R.H., Salunkea B.K. (2009). Production of Bioflocculant Exopolysaccharide by Bacillus Subtilis. Biotechnol. Adv..

[22] Fusconi R., Godinho M.J.L. (2002). Screening for Exopolysaccharide-Producing Bacteria from Sub-Tropical Polluted Groundwater. Braz. J. Biol..

[23] Kodali V.P., Sen R. (2008). Antioxidant and Free Radical Scavenging Activities of an Exopolysaccharide from a Probiotic Bacterium. Biotechnol. J..

[24] Ye S., Liu F., Wang J., Wang H., Zhang M. (2012). Antioxidant Activities of an Exopolysaccharide Isolated and Purified from Marine Pseudomonas PF-6. Carbohydr. Polym..

[25] El-Newary S.A., Ibrahim A.Y., Asker M.S., Mahmoud M.G., El Awady M.E. (2017). Production, Characterization and Biological Activities of Acidic Exopolysaccharide from Marine Bacillus Amyloliquefaciens 3MS 2017. Asian Pac. J. Trop. Med..

[26] Selim M.S., Amer S.K., Mohamed S.S., Mounier M.M., Rifaat H.M. (2018). Production and Characterisation of Exopolysaccharide from Streptomyces Carpaticus Isolated from Marine Sediments in Egypt and Its Effect on Breast and Colon Cell Lines. J. Genet. Eng. Biotechnol..

[27] Sun C., Wang J.-W., Fang L., Gao X.-D., Tan R.-X. (2004). Free Radical Scavenging and Antioxidant Activities of EPS2, an Exopolysaccharide Produced by a Marine Filamentous Fungus Keissleriella Sp. YS 4108. Life Sci..

[28] Asker M.M.S., Ahmed Y.M., Ramadan M.F. (2009). Chemical Characteristics and Antioxidant Activity of Exopolysaccharide Fractions from Microbacterium Terregens. Carbohydr. Polym..

[29] Di W., Zhang L., Yi H., Han X., Zhang Y., Xin L. (2018). Exopolysaccharides Produced by Lactobacillus Strains Suppress HT-29 Cell Growth via Induction of G0/G1 Cell Cycle Arrest and Apoptosis. Oncol. Lett..

[30] Wu J., Zhang Y., Ye L., Wang C. (2021). The Anti-Cancer Effects and Mechanisms of Lactic Acid Bacteria Exopolysaccharides in Vitro: A Review. Carbohydr. Polym..

[31] Hassan A.N., Ipsen R., Janzen T., Qvist K.B. (2003). Microstructure and Rheology of Yogurt Made with Cultures Differing Only in Their Ability to Produce Exopolysaccharides. Int. J. Dairy Sci..

[32] Ooi V.E., Liu F. (2000). Immunomodulation and Anti-Cancer Activity of Polysaccharide-Protein Complexes. Curr. Med. Chem..

[33] Peng Y., Zhang L., Zeng F., Kennedy J.F. (2005). Structure and Antitumor Activities of the Water-Soluble Polysaccharides from Ganoderma Tsugae Mycelium. Carbohydr. Polym..

[34] Wasser S.P. (2002). Medicinal Mushrooms as a Source of Antitumor and Immunomodulating Polysaccharides. Appl. Microbiol. Biotechnol..

[35] Li S., Xiong Q., Lai X., Li X., Wan M., Zhang J., Yan Y., Cao M., Lu L., Guan J. (2016). Molecular Modification of Polysaccharides and Resulting Bioactivities. Compr. Rev. Food Sci..

[36] Li S., Shah N.P. (2016). Characterization, Anti-Inflammatory and Antiproliferative Activities of Natural and Sulfonated Exo-Polysaccharides from Streptococcus Thermophilus ASCC 1275. J. Food Sci..

[37] Wang K., Li W., Rui X., Chen X., Jiang M., Dong M. (2014). Structural Characterization and Bioactivity of Released Exopolysaccharides from Lactobacillus Plantarum 70810. Int. J. Biol. Macromol..

[38] Sun N., Liu H., Liu S., Zhang X., Chen P., Li W., Xu X., Tian W. (2018). Purification, Preliminary Structure and Antitumor Activity of Exopolysaccharide Produced by Streptococcus Thermophilus CH9. Molecules.

[39] Li W., Tang W., Ji J., Xia X., Rui X., Chen X., Jiang M., Zhou J., Dong M. (2015). Characterization of a Novel Polysaccharide with Anti-Colon Cancer Activity from Lactobacillus Helveticus MB2-1. Carbohydr. Res..

[40] Wang J., Zhao X., Yang Y., Zhao A., Yang Z. (2015). Characterization and Bioactivities of an Exopolysaccharide Produced by Lactobacillus Plantarum YW32. Int. J. Biol. Macromol..

[41] Zarghi A., Arfaei S. (2011). Selective COX-2 Inhibitors: A Review of Their Structure-Activity Relationships. Iran J. Pharm. Res..

[42] Attiq A., Jalil J., Husain K., Ahmad W. (2018). Raging the War Against Inflammation With Natural Products. Front. Pharmacol..

[43] Jenab A., Roghanian R., Emtiazi G. (2020). Bacterial Natural Compounds with Anti-Inflammatory and Immunomodulatory Properties (Mini Review). Drug Des. Devel. Ther..

[44] Hayakawa M., Nonomura H. (1987). Humic Acid-Vitamin Agar, A New Medium for the Selective Isolation of Soil Actinomycetes. J. Ferment. Technol..

[45] Kim S.-W., Ahn S.-G., Seo W.-T., Kwon G.-S., Park Y.-H. (1998). Rheological Properties of a Novel High Viscosity Polysaccharide, A49-Pol, Produced by Bacillus Polymyxa. J. Microbiol. Biotechnol..

[46] Bergey D.H., Holt J.G., Hensyl W.R. (1994). Bergey’s Manual of Determinative Bacteriology.

[47] Tamura K., Peterson D., Peterson N., Stecher G., Nei M., Kumar S. (2011). MEGA5: Molecular Evolutionary Genetics Analysis Using Maximum Likelihood, Evolutionary Distance, and Maximum Parsimony Methods. Mol. Biol..

[48] Gardes M., Bruns T.D. (1993). ITS Primers with Enhanced Specificity for Basidiomycetes--Application to the Identification of Mycorrhizae and Rusts. Mol. Ecol..

[49] Liu C., Lu J., Lu L., Liu Y., Wang F., Xiao M. (2010). Isolation, Structural Characterization and Immunological Activity of an Exopolysaccharide Produced by Bacillus Licheniformis 8-37-0-1. Bioresour. Technol..

[50] Wang H., Jiang X., Mu H., Liang X., Guan H. (2007). Structure and Protective Effect of Exopolysaccharide from P. Agglomerans Strain KFS-9 against UV Radiation. Microbiol. Res..

[51] Nicely W.B., Wolfrom M.L., Tipson R.S. (1957). Infrared Spectra of Carbohydrates. Advances in Carbohydrate Chemistry and Biochemistry.

[52] Filisetti-Cozzi T.M., Carpita N.C. (1991). Measurement of Uronic Acids without Interference from Neutral Sugars. Anal. Biochem..

[53] Dodgson K.S., Price R.G. (1962). A Note on the Determination of the Ester Sulphate Content of Sulphated Polysaccharides. Biochem. J..

[54] Randall R.C., Phillips G.O., Williams P.A. (1988). The Role of the Proteinaceous Component on the Emulsifying Properties of Gum Arabic. Food Hydrocoll..

[55] You J., Dou L., Yoshimura K., Kato T., Ohya K., Moriarty T., Emery K., Chen C.-C., Gao J., Li G. (2013). A Polymer Tandem Solar Cell with 10.6% Power Conversion Efficiency. Nat. Commun..

[56] Brand-Williams W., Cuvelier M.E., Berset C. (1995). Use of a Free Radical Method to Evaluate Antioxidant Activity. LWT—Food Sci. Technol..

[57] Ruch R.J., Crist K.A., Klaunig J.E. (1989). Effects of Culture Duration on Hydrogen Peroxide-Induced Hepatocyte Toxicity. Toxicol. Appl. Pharm..

[58] Mosmann T. (1983). Rapid Colorimetric Assay for Cellular Growth and Survival: Application to Proliferation and Cytotoxicity Assays. J. Immunol. Methods.

[59] Granica S., Czerwińska M.E., Piwowarski J.P., Ziaja M., Kiss A.K. (2013). Chemical Composition, Antioxidative and Anti-Inflammatory Activity of Extracts Prepared from Aerial Parts of *Oenothera Biennis* L. and *Oenothera Paradoxa* Hudziok Obtained after Seeds Cultivation. J. Agric. Food Chem..

[60] Amessis-Ouchemoukh N., Madani K., Falé P.L.V., Serralheiro M.L., Araújo M.E.M. (2014). Antioxidant Capacity and Phenolic Contents of Some Mediterranean Medicinal Plants and Their Potential Role in the Inhibition of Cyclooxygenase-1 and Acetylcholinesterase Activities. Ind. Crop. Prod..

[61] Petrovic N., Murray M. (2010). Using *N,N,N’,N’*-Tetramethyl-*p*-Phenylenediamine (TMPD) to Assay Cyclooxygenase Activity in Vitro. Methods Mol. Biol..

[62] Shinde U.A., Phadke A.S., Nair A.M., Mungantiwar A.A., Dikshit V.J., Saraf M.N. (1999). Membrane Stabilizing Activity—A Possible Mechanism of Action for the Anti-Inflammatory Activity of Cedrus Deodara Wood Oil. Fitoterapia.

[63] Lehmann P.F., Murray P.R., Baron E.J., Pfaller M.A., Tenover F.C., Yolken R.H. (1999). Manual of Clinical Microbiology. Mycopathologia.

